# Supplementary material for: Targeting MHC Regulation Using Polycyclic Polyprenylated Acylphloroglucinols Isolated from Garcinia bancana
Source: Biomolecules. 2020 Sep 2;10(9):1266. doi: 10.3390/biom10091266 (PMC7563419; doi:10.3390/biom10091266)
Supplement: Supplementary file 1 [file biomolecules-10-01266-s001.pdf]

Supporting information

Targeting MHC regulation using polycyclic polyprenylated acylphloroglucinols isolated from Garcinia *bancana*

Chloé Coste,[1,2] Nathalie Gérard,[1] Antoine Bruguière,[2] Chau Phi Dinh,[2] Caroline Rouger,[2,†] Sow Tein Leong,[3] Khalijah Awang,[3] Pascal Richomme,[2] Séverine Derbré,[2,*] Béatrice Charreau[1,*]

[1]Université de Nantes, CHU Nantes, Inserm, Centre de Recherche en Transplantation et en Immunologie, UMR 1064, ITUN, F-44000 Nantes, France.

[2]SONAS, EA921, UNIV Angers, SFR QUASAV, Faculty of Health Sciences, Dpt Pharmacy, 49045 Angers cedex 01, France.

[3]Department of Chemistry, Faculty of Science, University of Malaya, Kuala Lumpur, Malaysia

*Both last authors contributed equally to the work as senior authors

†Current address: Unité de recherche Œnologie, EA 4577, USC 1366 INRAE, ISVV, Université de Bordeaux, 33882 Villenave d'Ornon, France

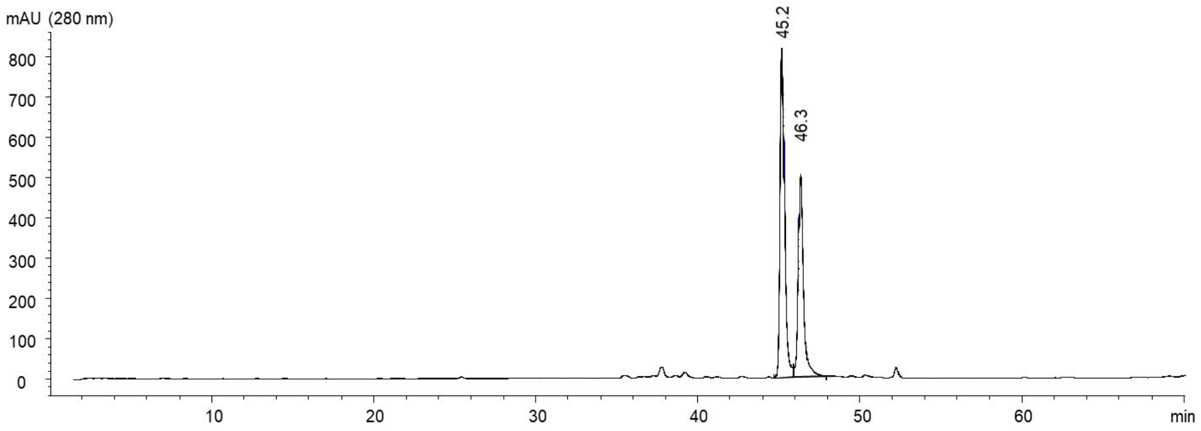

Figure S1. HPLC-UV (λ_{max} 280 nm) chromatogram of **GX** from *Garcina bancana* (bark) dichloromethanic extract (Batch KL4967). Guttiferone F (**3**, t_R = 45.2 min) represents 59.6% and xanthochymol (**2**, t_R = 46.3 min) 40.4% at 280 nm.

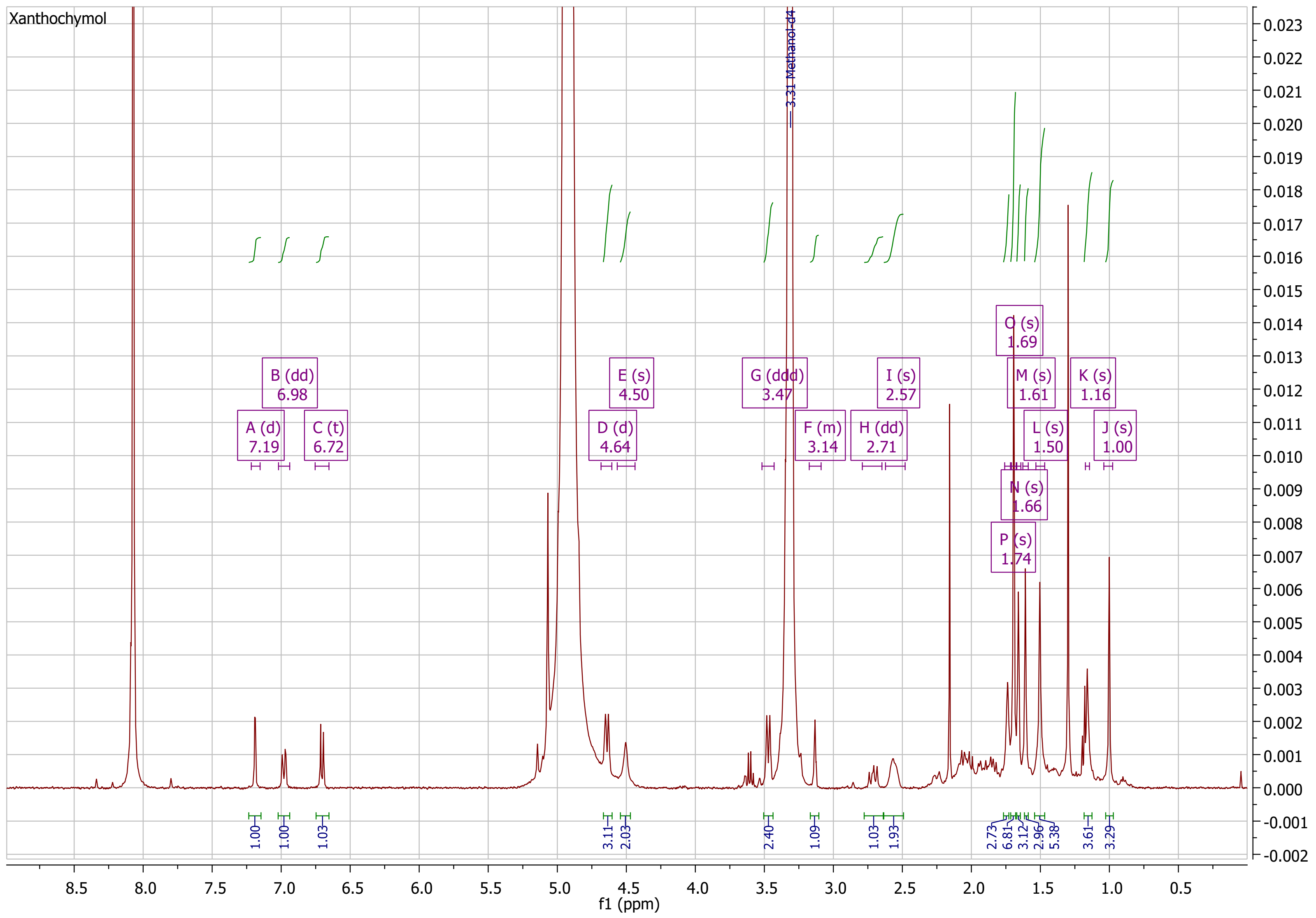

Figure S2. ^{1}H-NMR spectrum of xanthochymol **2** recorded in methanol-d4 +0.1% deuterated TFA at 400 MHz.

Supporting information

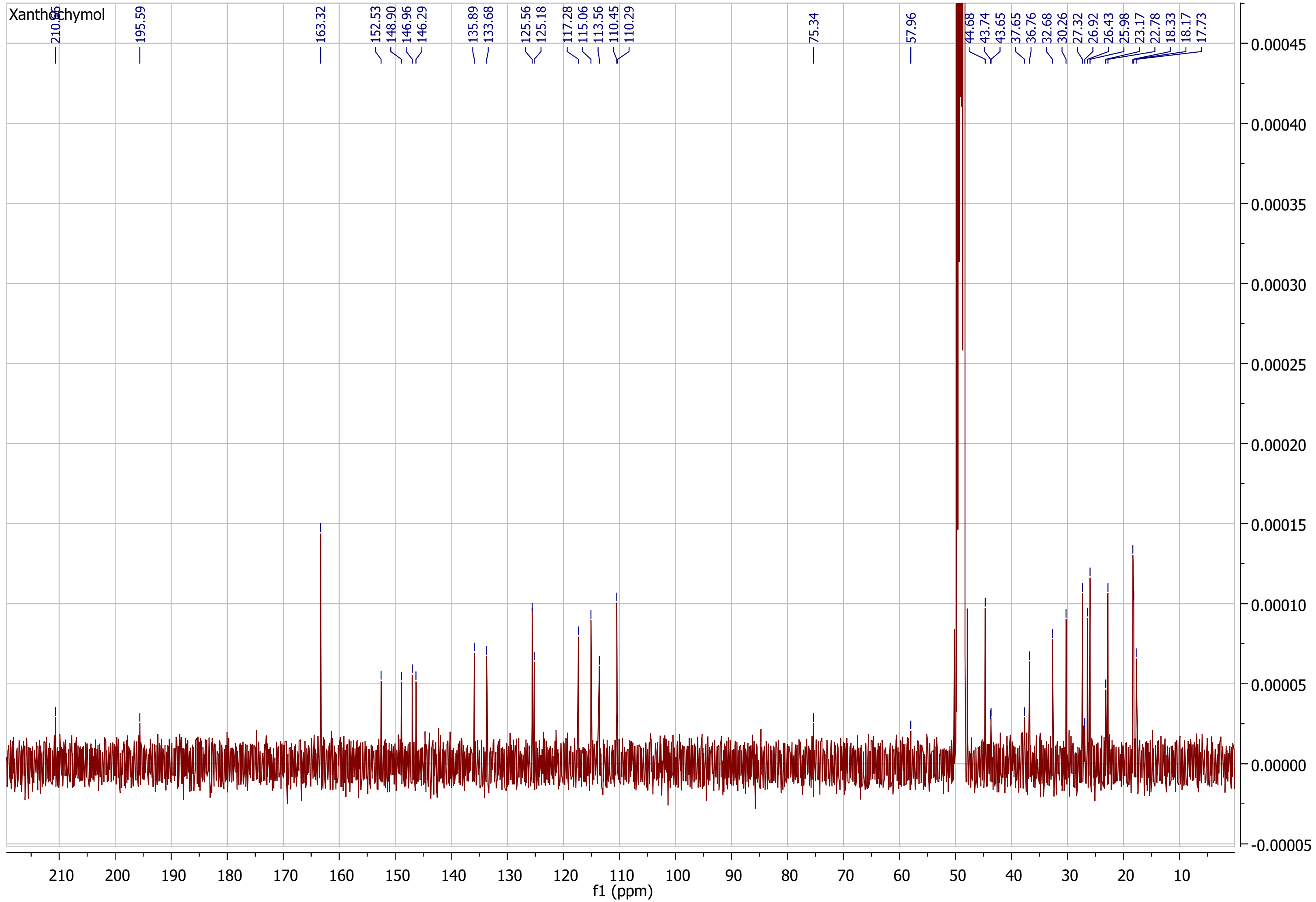

Figure S3. ^{13}C-NMR spectrum of xanthochymol **2** recorded in methanol-d4 +0.1% deuterated TFA at 100 MHz.

Supporting information

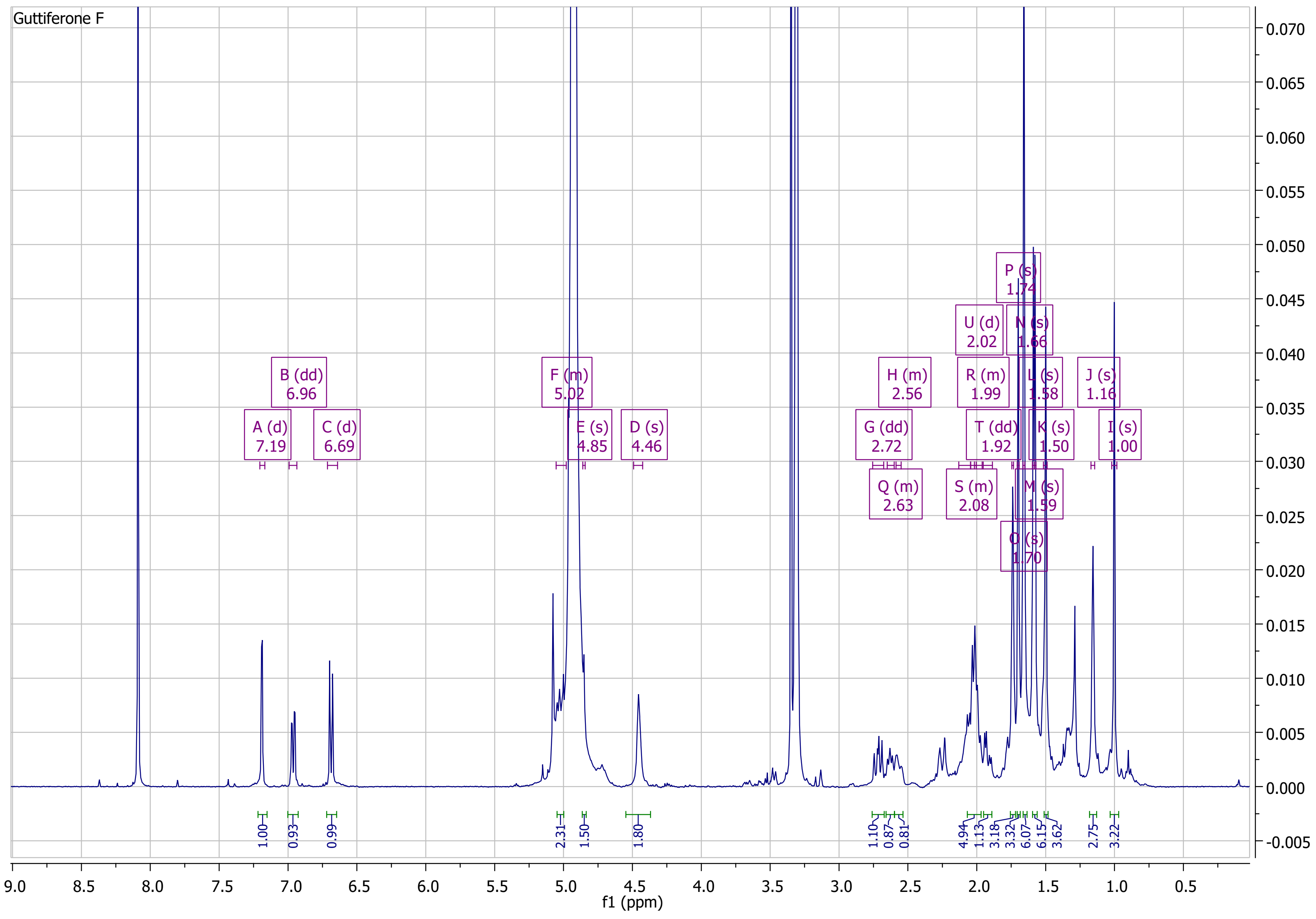

Figure S4. ^{1}H-NMR spectrum of guttiferone F **3** recorded in methanol-d4 +0.1% deuterated TFA at 400 MHz.

Supporting information

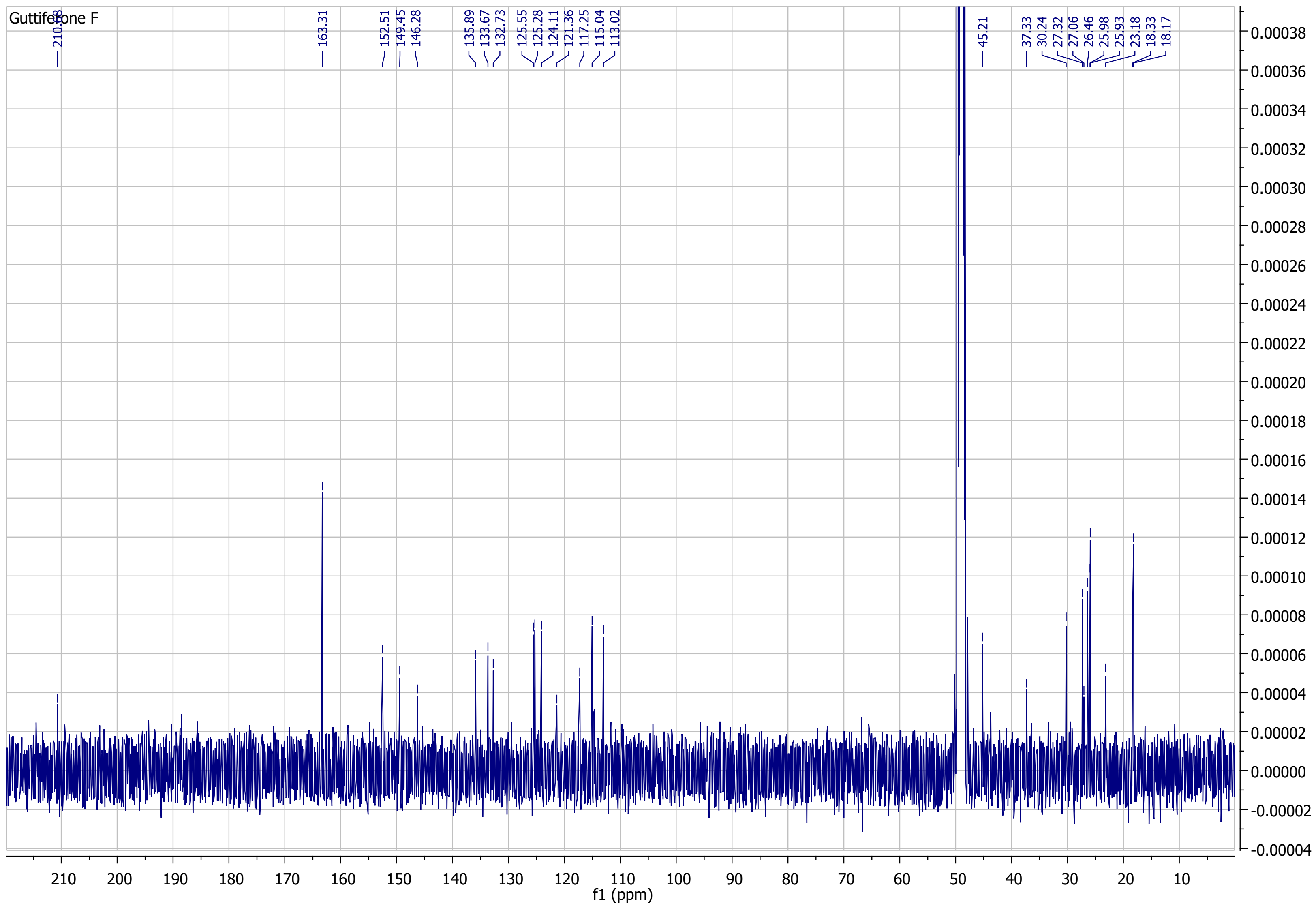

Figure S5. ^{13}C-NMR spectrum of guttiferone F **3** recorded in methanol-d4 +0.1% deuterated TFA at 100 MHz.

Confluent EC monolayers were incubated with diluent only as a negative control, simvastatine or zoledronic acid (ZA) at 10 μM in the absence (unstimulated endothelial cells) or in the presence (+ IFNγ) of IFNγ (100 U/mL) for 48 h. Cells were harvested, subjected to immunolabeling with specific antibodies against HLA class I, HLA class II, HLA-E and MICA and analyzed by flow cytometry. Data are depicted as histograms of fluorescence intensity (x-axis) versus cell number (y-axis) for MHC molecules (red) and for controls (irrelevant isotype control antibodies, grey). Geometric means of fluorescence are indicated in red.

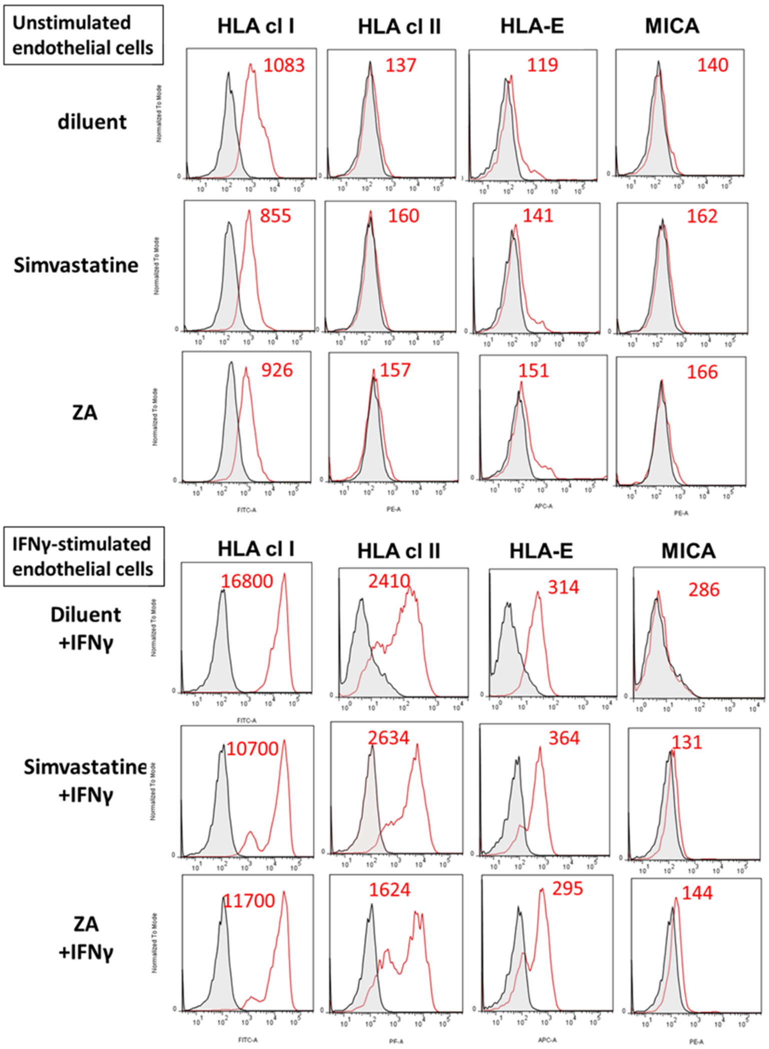

Figure S6. Effect of Simvastatine and Zoledronic acid (ZA) on the expression of MHC molecules.

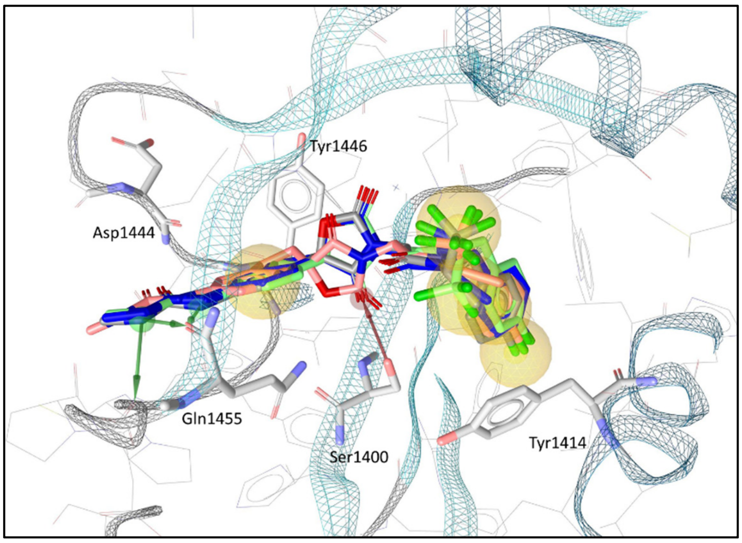

Figure S7. Superimposes of the original structure A-485 and its energy-minimized conformation as well as the rebuilt structure in the binding site of histone acetyltransferase p300. Red and green arrows represent hydrogen bond acceptors and donors, respectively; and yellow spheres show the hydrophobic contacts between the ligands and the protein.

Supporting information

Table S1. *Garcinia* species from Malaysia (name, voucher number and available organs) and data on their phytochemistry.

Garcinia species	**Voucher number** (KL, Kuala Lumpur) and **organs** (**B** = bark, **L** = leaf, **Fr** = fruit)	Data on phytochemistry	
		Secondary metabolites previously described	Presence of PPAPs as major products in DCM extracts according to LC-UV-MS²*
G. bancana Miq.	**4967** (B, L, Fr), **5033** (B, L)	Biphenyls, PPAPs, flavonoids, triterpènes [1]	B and L: Yes
G. cowa Roxb.	**4587**, **5526** (B, L)	Biphenyls, xanthones, flavonoids, terpenes [2]	NA
G. diversifolia King	**5712** (B, L)	Triterpenes [3]	NA
G. dumosa King	**5516** (B, L)	-	No (undetermined)
G. brevirostris Scheff. (= *G. eugeniaefolia* Wall.)	**4611**, **5074**, **5273**, **5456** (B, L), **5251** (B)	Benzophenones [4]	No (Tocotrienols)
G. forbesii King	**5146**, **5518** (B, L)	Xanthones [5]	NA
G. gaudichaudii Planch. & Triana	**5483** (B, L, Fr)	Xanthones [6]	NA
G. griffithii T. Anderson	**5303** (B, L)	Xanthones, PPAPs [7]	B: No (Xanthones)<br>L: Yes
G. celebica L. (= *G. hombroniana* Pierre)	**4800** (B, L)	Triterpenes, xanthones, benzophenones, flavonoids [8]	No (Triterpenes)
G. nervosa Miq.	**5702** (B, L)	Flavonoids [9]	NA
G. nigrolineata Planch.	**5555** (B, L)	Biphenyls, xanthones [10]	NA
G. opaca King	**4532** (B, L, Fr), **4560** (B, L)	Xanthones [11], terpenes [12]	NA
G. opaca King **var. dumosa** Whitmore[a]	**5589** (B, L)		NA
G. parvifolia (Miq.) Miq.	**5073**, **5259** (B, L), **5248** (B), **5670** (B, L, Fr)	Flavonoids [13], phloroglucinols, depsidones, xanthones [14]	No (Xanthones)
G. prainiana King	**5661** (B, L)	Triterpenes, flavonoids [15]	NA
G. pyrifera Ridl.	**4523**, **4954**, **5443** (B, L)	PPAPs (fruits), xanthones (bark) [16]	NA
G. rostrata (Hassk.) Miq.	**5175** (B, L)	Xanthones [17]	NA

* Several products with λ_{max} around 230-235, 275-280 and 320 nm and a molecular weight between 560 and 618 Da [18]. NA : Not analyzed.

Table S2. ChemPLP scores for the top 10 poses in docking experiments.

Native ligand A-485	Guttiferone J (1)	Xanthochymol (2)	Guttiferone F (3)	Garcinol (4)
114.36	96.35	82.46	90.77	88.04
109.57	86.53	79.35	89.83	87.19
107.02	71.77	78.44	86.36	86.52
84.45	70.04	69.06	85.77	86.01
	69.41		83.31	84.63
	69.17		76.01	84.25
	68.04		74.99	83.87
	64.25		72.63	82.75
	64.21		71.99	81.23
	62.14		71.76	80.19

The cut-off cluster of docking poses was defined at 0.5 Å. For the energy-minimized reconstructed ligand (A-485) and xanthochymol, only four docked poses were obtained for each compound. This means other conformations were clustered in these best-docked poses.

References

1. Rukachaisirikul, V.; Naklue, W.; Sukpondma, Y.; Phongpaichit, S., An antibacterial biphenyl derivative from *Garcinia bancana* Miq. *Chemical & Pharmaceutical Bulletin* **2005,** *53* (3), 342-343.
2. Siridechakorn, I.; Maneerat, W.; Sripisut, T.; Ritthiwigrom, T.; Cheenpracha, S.; Laphookhieo, S., Biphenyl and xanthone derivatives from the twigs of a *Garcinia* sp (*Clusiaceae*). *Phytochemistry Letters* **2014,** *8*, 77-80.
3. Tay, B. Y. P. Chemical constituents of *Garcinia mangostana, G. parvifolia, G. griffithi* and *G. diversifolia* (*Guttiferae*) and their biological activities. Science Master thesis, Universiti Putra Malaysia, Serdang, 1996.
4. Hartati, S.; Soemiati, A.; Wang, H.-B.; Kardono, L. B. S.; Hanafi, M.; Kosela, S.; Qin, G.-W., A novel polyisoprenyl benzophenone derivative from *Garcinia eugeniaefolia*. *Journal of Asian Natural Products Research* **2008,** *10* (6), 509-513.
5. Leong, Y. W.; Harrison, L. J.; Bennett, G. J.; Tan, H. T. W., Forbesione, a modified xanthone from *Garcinia forbesii*. *Journal of Chemical Research-S* **1996,** (8), 392-393.
6. Xu, Y. J.; Yip, S. C.; Kosela, S.; Fitri, E.; Hana, M.; Goh, S. H.; Sim, K. Y., Novel cytotoxic, polyprenylated heptacyclic xanthonoids from Indonesian *Garcinia gaudichaudii* (Guttiferae). *Organic Letters* **2000,** *2* (24), 3945-3948.
7. Nilar; Nguyen, L.-H. D.; Venkatraman, G.; Sim, K.-Y.; Harrison, L. J., Xanthones and benzophenones from *Garcinia griffithii* and *Garcinia mangostana*. *Phytochemistry* **2005,** *66*, 1718-1723.
8. Jamila, N.; Khairuddean, M.; Khan, S. N.; Khan, N.; Osman, H., Phytochemicals from the bark of *Garcinia hombroniana* and their biological activities. *Records of Natural Products* **2014,** *8*, 312-316.
9. Ilyas, M.; Kamil, M.; Parveen, M.; Khan, M. S., Isoflavones from *Garcinia nervosa*. *Phytochemistry* **1994,** *36* (3), 807-809.
10. Rukachaisirikul, V.; Tadpetch, K.; Watthanaphanit, A.; Saengsanae, N.; Phongpaichit, S., Benzopyran, Biphenyl, and tetraoxygenated xanthone derivatives from the twigs of *Garcinia nigrolineata*. *Journal of Natural Products* **2005,** *68*, 1218-1221.
11. Goh, S. H.; Jantan, I.; Gray, A. I.; Waterman, P. G., Prenylated xanthones from *Garcinia opaca*. *Phytochemistry* **1992,** *31*, 1383-1386.
12. Mori, R.; Nugroho, A. E.; Hirasawa, Y.; Wong, C. P.; Kaneda, T.; Shirota, O.; Hadi, A. H. A.; Morita, H., Opaciniols A-C, new terpenoids from *Garcinia opaca*. *Journal of Natural Medicines* **2014,** *68*, 186-191.
13. Hassan, S. H. A.; Fry, J. R.; Abu Bakar, M. F., Phytochemicals content, antioxidant activity and acetylcholinesterase inhibition properties of indigenous *Garcinia parvifolia* fruit. *BioMed Research International* **2013,** *2013*, 138950.
14. Rukachaisirikul, V.; Naklue, W.; Phongpaichit, S.; Towatana, N. H.; Maneenoon, K., Phloroglucinols, depsidones and xanthones from the twigs of *Garcinia parvifolia*. *Tetrahedron* **2006,** *62*, 8578-8585.
15. Klaiklay, S.; Sukpondma, Y.; Rukachaisirikul, V.; Hutadilok-Towatana, N.; Chareonrat, K., Flavanone glucuronides from the leaves of *Garcinia prainiana*. *Canadian Journal of Chemistry* **2011,** *89*, 461-464.
16. Roux, D.; Hadi, H. A.; Thoret, S.; Guenard, D.; Thoison, O.; Pais, M.; Sevenet, T., Structure-activity relationship of polyisoprenyl benzophenones from *Garcinia pyrifera* on the tubulin/microtubule system. *Journal of Natural Products* **2000,** *63* (8), 1070-1076.
17. Wong, K. W.; Ee, G. C. L.; Ismail, I. S.; Jong, V. Y. M., Xanthones from Stem Bark of Garcinia rostrata. *Chemistry of Natural Compounds* **2018,** *54* (6), 1160-1163.
18. Rouger, C. Activité pharmacologique de dérivés polyphénoliques isolés de Clusiaceae et de Calophyllaceae malaisiennes : effets régulateurs sur des marqueurs endothéliaux de l'inflammation et de l'immunité. PhD thesis, University of Angers, 2015.

Supporting information

Abbreviations:

CIITA pIV: class II transactivator promotor IV
CBP: CREB binding protein
CREB: cAMP-responsive element binding protein
CsA: Cyclosporine A
DCM: dichloromethane
EC: endothelial cell
HAT: histone acetyltransferase
HDAC: histone deacetylase
HLA: human leukocyte antigen
IFNγ: interferon γ
IFNGR: IFNγ receptor
IRF9: IFN regulatory factor 9
JAK: Janus kinase
K: lysine
MHC: major histocompatibility complex
MICA: MHC class I-related chain A
NPs: natural products
PPAPs: polycyclic polyprenylated acylphloroglucinols
SAHA: suberoylanilide hydroxamic acid
Sim: simvastatin
SOCS: suppressor of cytokine signaling
STAT : signal transducer and activator of transcription
TNF: tumor necrosis factor
TSA: trichostatin A
Y: tyrosine
ZA: zoledronic acid
